# Early endocrine, bone, and inflammatory responses to microwave ablation for hyperparathyroidism: preliminary study

**DOI:** 10.3389/fendo.2026.1763008

**Published:** 2026-07-07

**Authors:** Ying Wei, Zhenlong Zhao, Jie Wu, Shiliang Cao, Na Yu, Wenjia Cai, Yan Li, Lili Peng, Ming’an Yu

**Affiliations:** Department of Interventional Medicine, China-Japan Friendship Hospital, Beijing, China

**Keywords:** bone remodeling, hyperparathyroidism, inflammation mediators, microwave ablation, parathyroid hormone

## Abstract

**Purpose:**

To investigate short-term changes in hormonal, bone metabolic, and inflammatory markers after ultrasound-guided microwave ablation (MWA) in patients with hyperparathyroidism (HPT).

**Methods:**

Nineteen patients (14 with primary HPT[PHPT], 5 with secondary HPT[SHPT]) underwent MWA. Serum intact parathyroid hormone (iPTH), calcium, phosphate, alkaline phosphatase (ALP), bone turnover markers [e.g., procollagen type I N-terminal propeptide (PINP), bone-specific ALP (BALP), fibroblast growth factor-23 (FGF-23), etc.], and inflammatory markers [e.g., C-reactive protein (CRP), interleukin-6 (IL-6), tumor necrosis factor-α (TNF-α), etc.] were measured at baseline, day 1, and month 1 post-ablation.

**Results:**

iPTH and calcium decreased by day 1 (both P < 0.001); iPTH remained reduced at month 1 (P < 0.001), while calcium slightly increased (P = 0.046). Phosphate showed a borderline upward trend (P = 0.050), and ALP decreased at month 1 (P = 0.044). PINP increased at month 1 (P = 0.011; day 1 to month 1 P = 0.001), BALP trended upward (P = 0.112), and FGF-23 declined over time (P = 0.015). CRP peaked at day 1 and decreased by month 1 (P = 0.036); other cytokines remained unchanged. In PHPT, iPTH and calcium decreased on day 1 (both P ≤ 0.001), PINP increased by month 1 (P = 0.013), and FGF-23 declined (P = 0.009). In SHPT, trends were directionally similar but not statistically significant; calcium decreased on day 1 (P = 0.043) and rebounded by month 1. No severe hypocalcemia occurred; two patients experienced transient hoarseness.

**Conclusion:**

Microwave ablation induces rapid biochemical normalization and early skeletal and inflammatory changes in patients with hyperparathyroidism.

## Introduction

Hyperparathyroidism (HPT) is a common endocrine disorder characterized by inappropriate secretion of parathyroid hormone (PTH), leading to disturbances in calcium and phosphate metabolism (1). It is generally classified into primary HPT (PHPT), which predominantly affects elderly individuals and postmenopausal women (2), and secondary HPT (SHPT), which frequently occurs in patients with end-stage renal disease, particularly those undergoing hemodialysis (3, 4). The prevalence of SHPT in dialysis populations can exceed 60% (5, 6).

In recent years, ultrasound-guided percutaneous microwave ablation (MWA) has emerged as a promising minimally invasive treatment for HPT (7–12). Accumulating evidence supports the efficacy of MWA in both PHPT and SHPT, with reported benefits such as significant reductions in serum PTH and calcium levels, symptomatic improvement, and a low incidence of complications (11–14). Correspondingly, expert consensus statements have begun to define its indications, efficacy, and safety, highlighting its growing role in the management of HPT (15).

Beyond biochemical correction, HPT is increasingly recognized as a systemic condition involving altered bone metabolism and low-grade inflammation (1, 16, 17). While symptom relief after MWA has been widely observed, it remains unclear whether these clinical improvements are also accompanied by changes in systemic inflammatory status. Previous studies have examined inflammatory and bone turnover markers following parathyroidectomy (18, 19), but evidence remains limited regarding the immunometabolic effects of MWA. Therefore, this prospective observational study was designed to evaluate early changes in parathyroid-related hormones, bone metabolic markers, and inflammatory mediators after MWA. By analyzing dynamic alterations at baseline, 1 day, and 1 month post-ablation, the study aims to provide preliminary insights into the systemic biological responses to MWA and its potential relevance to treatment evaluation and long-term outcomes.

## Methods and materials

### Patients

This prospective observational study was conducted at the Department of Interventional Medicine, China-Japan Friendship Hospital. From January 2023 to January 2025, 19 consecutive patients diagnosed with HPT underwent ultrasound-guided percutaneous MWA. Among them, 14 patients had PHPT and 5 had SHPT. All participants provided written informed consent for both the treatment and study participation prior to enrollment. This study was prospectively registered in the Chinese Clinical Trial Registry (ChiCTR1900020680).

### Inclusion and exclusion criteria

#### Inclusion criteria

For PHPT patients:

i) Symptomatic PHPT; ii) Asymptomatic PHPT meeting any of the following: serum calcium > upper normal limit by ≥ 0.25 mmol/L; evidence of bone and/or kidney involvement (creatinine clearance < 60 mL/min; T-score < –2.5 at the lumbar spine, total hip, femoral neck, or distal one-third radius; and/or presence of fragility fracture); or iii) Lack of response to pharmacological therapy (20).

For SHPT patients:

i) End-stage renal disease with drug-refractory SHPT; ii) Continuous iPTH ≥ 500 pg/mL despite adequate dialysis and medical management, or iPTH < 500 pg/mL with uncontrolled hypercalcemia/hyperphosphatemia; or iii) Presence of clinical manifestations attributable to SHPT (3, 15).

Common to both PHPT and SHPT:

i) At least one suspicious HPT lesion on ultrasound with typical benign characteristics and maximum diameter ≥ 0.6 cm (21); ii) Positive ^99^mTc-MIBI uptake in both early and delayed phases, or negative radionuclide scan combined with typical ultrasound characteristics; iii) Ultrasound evaluation confirming a safe and feasible puncture path; iv) Refusal of or ineligibility for surgery.

#### Exclusion criteria

i) Mental or consciousness disorders preventing cooperation;

ii) Severe coagulopathy or inadequate withdrawal from anticoagulants; iii) Imaging findings strongly suggestive of malignancy; iv) Unwillingness or inability to complete scheduled follow-up; v) History of neck surgery with significant anatomical distortion precluding safe ablation.

SHPT was defined as persistent PTH elevation caused by chronic kidney disease or other reversible factors, occurring before parathyroid hyperplasia progresses. Tertiary hyperparathyroidism (THPT) was defined as persistent PTH elevation continuing after renal transplantation or correction of the underlying cause, usually associated with autonomous parathyroid hyperplasia. Patients meeting these criteria were classified accordingly. Among the 19 enrolled patients, all SHPT patients had end-stage renal disease on dialysis, and none had received renal transplantation; therefore, no THPT patients were included in this cohort.

### Equipment and procedures

B-mode ultrasound (US), contrast-enhanced US, and MWA were performed using a LOGIQ E9 system (GE Healthcare, USA) equipped with a 9.0-MHz linear-array transducer. A 17G internally cooled microwave antenna with a 3-mm active tip (Intelligent Basic Type Microwave Tumor Ablation System, Nanjing ECO Microwave System, China) was used for ablation. The US contrast agent was Sonazoid (Daiichi-Sankyo, Tokyo, Japan). All procedures were performed by three radiologists, each with more than 5 years of experience in thermal ablation for HPT. A fascial space–based hydrodissection technique was applied to separate the target gland from adjacent critical structures (22). Pre-procedural assessment and MWA were performed as previously described (13). All HPT lesions identified by US were targeted for complete ablation in a single session whenever feasible. Lesions that were excessively large were treated in planned staged sessions; if unilateral recurrent laryngeal nerve injury occurred, ablation of the contralateral lesion was deferred to a subsequent session.

### Laboratory measurements

Parathyroid-related biochemical parameters, including intact parathyroid hormone (iPTH), serum calcium, serum phosphate, and alkaline phosphatase (ALP), were measured at baseline, 1 day, and 1 month after MWA.

For bone metabolism and inflammatory markers, venous blood samples were collected at baseline, 1 day, and 1 month after MWA. Serum was separated and stored at −70 °C until batch analysis, and all submitted samples were tested in the same batch. Bone metabolism markers included pyridinoline (PYD), bone-specific alkaline phosphatase (BALP), procollagen type I N-terminal propeptide (PINP), C-terminal telopeptide of type I collagen (CTX-I), tartrate-resistant acid phosphatase 5b (TRACP-5b), osteoprotegerin (OPG), osteocalcin (OT-BGP), and fibroblast growth factor-23 (FGF-23). Inflammatory markers included interleukin-12p70 (IL-12p70), interleukin-6 (IL-6), tumor necrosis factor-α (TNF-α), interferon-γ (IFN-γ), and C-reactive protein (CRP). All assays were performed using commercially available enzyme-linked immunosorbent assay (ELISA) kits (TechLab, China) according to the manufacturer’s protocols, employing a double-antibody sandwich method. Optical density values were determined with a microplate reader, and concentrations were calculated from standard curves.

All biochemical markers, especially bone metabolism and inflammatory markers, were interpreted in the context of renal function, as chronic kidney disease can influence baseline levels and dynamics of these biomarkers.

### Follow-up and outcomes

Follow-up evaluations were conducted at 1 day and 1 month post-ablation, including clinical assessment, laboratory testing, and US examination. The primary outcome was early change in parathyroid-related biochemical parameters (iPTH, calcium, phosphate, and ALP following MWA. Secondary outcomes included early changes in bone metabolism markers (PYD, BALP, PINP, CTX-I, TRACP-5b, OPG, OT-BGP, and FGF-23) and inflammatory markers (IL-12p70, IL-6, TNF-α, IFN-γ, and CRP).

### Statistical analysis

All statistical analyses were conducted using IBM SPSS Statistics (version 16.0). Normally distributed data were presented as mean ± standard deviation (SD), and non-normally distributed data were expressed as median and interquartile range (IQR). Given the small sample size and the mixed distribution of variables, non-parametric tests were applied for comparisons across the three time points (baseline, day 1 post-ablation, and month 1 post-ablation). The Friedman test was used for overall comparisons, followed by Wilcoxon signed-rank tests for pairwise comparisons with Bonferroni correction. A two-sided P value <0.05 was considered statistically significant.

## Results

### Baseline characteristics

A total of 19 patients with HPT were enrolled, including 14 with PHPT and 5 with SHPT. The cohort consisted of 14 females and 5 males, with a mean age of 52.2 ± 15.0 years (range: 28-70). A total of 27 parathyroid lesions were treated. Lesion distribution included 3 in the left superior, 9 in the left inferior, 3 in the right superior, 11 in the right inferior, and 1 ectopic gland. The mean maximum diameter of the lesions was 1.5 ± 0.5 cm (range: 0.7-2.7cm), with a median volume of 0.381 mL (IQR: 0.176-1.791). The baseline median iPTH level was 172.8 pg/mL (IQR: 111.7-344.6), and the mean serum calcium level was 2.78 ± 0.19 mmol/L. Additional baseline clinical and procedural parameters are presented in **Table 1**.

**Table 1:** Baseline characteristics.

Parameters	Overall
Sex (Male/Female)	5/14
Age (years)^†^	52.2 ± 15.0
Body mass index (kg/m^2^)^†^	23.92 ± 3.68
PHPT/SHPT	14/5
No. of glands (n)	27
Lesion location (n)	
Left superior	3
Left inferior	9
Right superior	3
Right inferior	11
Ectopic	1
Maximum diameter (cm)^†^	1.5 ± 0.5
Volume (mL)^‡^	0.381 (0.176-1.791)
Ablation energy (KJ)^‡^	2.19 (0.29-11.73)
Hydrodissection Fluid Volume (mL)^‡^	70 (24-254)

* Statistically significant difference (P<0.05). † Data are means ± SDs. ‡ Data are medians, with IQRs in parentheses.

PHPT, primary hyperparathyroidism; SHPT, secondary hyperparathyroidism.

### Ablation procedures

All patients underwent ultrasound-guided percutaneous MWA according to the protocol. Eighteen patients completed the procedure in one session, while one patient with multiple parathyroid lesions underwent planned two-session ablation. The median ablation energy was 2.19 kJ (IQR: 0.29-11.73). Hydrodissection was performed in all cases, with a median hydrodissection fluid volume of 70 mL (24-254).

### Parathyroid-related laboratory parameters

At 1 day post-ablation, serum iPTH and calcium levels decreased significantly (both P < 0.001). Phosphate levels showed an upward trend (P = 0.050), though without statistical significance. ALP levels remained stable (P = 0.037). At 1 month post-ablation, iPTH levels partially rebounded but remained significantly lower than baseline (P < 0.001).Serum calcium levels increased slightly (P = 0.046). Phosphate levels continued to rise (P = 0.051). ALP levels slightly decreased (P = 0.044)(**Table 2**).

**Table 2:** Changes in parathyroid-related laboratory parameters before and after ablation.

Time point	iPTH (pg/mL)	Calcium (mmol/L)	Phosphorus (mmol/L)	ALP (U/L)
Pre-ablation	172.8 (111.7, 344.6)	2.78 ± 0.19	0.87 (0.82, 1.54)	102 (67-126)
Day 1 Post-ablation	28.3 (7.1, 78.4)	2.40 ± 0.23	1.08 (0.97, 1.50)	102 (67-110)
Month 1 Post-ablation	82.3 (60.6, 108.7)	2.56 ± 0.38	1.15 (1.01, 1.34)	82 (65-107)
P(overall)	<0.001*	<0.001*	0.050	0.037*
P1†	<0.001*	<0.001*	—	0.029*
P2‡	0.030*	0.069	—	0.546
P3§	<0.001*	0.046*	—	0.044*

†P1: Pre-ablation vs. Day 1 Post-ablation, ‡P2: Day 1 Post-ablation vs. Month 1 Post-ablation, §P3: Pre-ablation vs. Month 1 Post-ablation. *P < 0.05 is considered statistically significant.

ALP, alkaline phosphatase; iPTH, intact parathyroid hormone.

Subgroup analyses revealed that in PHPT patients, iPTH and calcium levels decreased on day 1 and remained reduced at month 1 (both P < 0.05). Phosphorus levels increased at month 1 (P = 0.016), while ALP remained stable throughout. In contrast, in SHPT patients, reductions in iPTH and phosphate were not statistically significant, although calcium significantly declined on day 1 (P = 0.043) and returned to near-baseline levels by month 1 (P = 0.043). ALP levels showed a progressive decline (P = 0.022) (**Supplementary Table 1**, **2**).

### Bone metabolism markers

PINP levels increased significantly at 1 month post-ablation (P = 0.011), with a marked increase between day 1 and month 1 (P = 0.001). BALP showed an upward trend from day 1 to month 1, although overall changes were not statistically significant (P = 0.112). FGF-23 levels decreased significantly during the follow-up period (P = 0.015), with significant reductions between day 1 and month 1 (P = 0.025), and between baseline and month 1 (P = 0.016). No significant changes were observed in other bone metabolism markers, including PYD, CTX-I, TRACP-5b, OPG, and OT-BGP (all P > 0.05) (**Table 3**).

**Table 3:** Changes in bone metabolism markers before and after ablation.

Time point	PINP (pg/mL)	BALP (ng/mL)	PYD (ng/mL)	CTX-I (ng/mL)	TRACP-5b (mIU/mL)	OPG (pg/mL)	OT-BGP (pg/mL)	FGF-23 (pg/mL)
Pre-ablation	13244.66 ± 15135.98	0.78 (0.37, 3.47)	10.99 (1.50, 32.81)	7.93 ± 4.87	4.22 ± 2.54	64.39 (26.52, 130.30)	8355.91 (2785.60-16136.10)	0.09 (0.01, 5.97)
Day 1 Post-ablation	12521.05 ± 11185.68	0.55 (0.37, 1.41)	1.50 (1.50, 12.04)	7.36 ± 5.41	3.76 ± 2.41	51.55 (8.97, 114.67)	4291.46 (2625.83-21297.23)	0.01 (0.01, 2.39)
Month 1 Post-ablation	22560.29 ± 6905.27	1.09 (0.66, 3.87)	1.50 (1.50, 7.99)	7.03 ± 5.21	3.19 ± 3.01	39.86 (8.97, 137.64)	7886.02 (999.85-16409.32)	0.01 (0.01, 0.01)
P(overall)	<0.001	0.112	0.052	0.879	0.411	0.186	0.065	0.015*
P1†	0.688	—	—	—	—	—	—	0.583
P2‡	0.001*	—	—	—	—	—	—	0.025*
P3§	0.011*	—	—	—	—	—	—	0.016*

†P1: Pre-ablation vs. Day 1 Post-ablation, ‡P2: Day 1 Post-ablation vs. Month 1 Post-ablation, §P3: Pre-ablation vs. Month 1 Post-ablation. *P < 0.05 is considered statistically significant.

PYD, pyridinoline; BALP, bone-specific alkaline phosphatase; PINP, procollagen type I N-terminal propeptide; CTX-I, C-terminal telopeptide of type I collagen; TRACP-5b, tartrate-resistant acid phosphatase 5b; OPG, osteoprotegerin; OT-BGP, osteocalcin; and FGF-23, fibroblast growth factor-23. Biomarker levels in SHPT patients may be influenced by renal function.

In PHPT patients, PINP levels increased significantly by month 1 (P = 0.013), particularly between day 1 and month 1 (P = 0.002), while FGF-23 showed a significant decline over time (P = 0.009). BALP displayed an upward trend from day 1 to month 1, though overall changes were not statistically significant. In SHPT patients, although none of the bone turnover markers reached statistical significance (all P > 0.05), PINP tended to increase, FGF-23 declined progressively, and BALP exhibited a mild downward trend (**Supplementary Table 3**, **4**).

### Inflammatory markers

CRP levels increased slightly at day 1 post-ablation (median 5201.34 pg/mL) compared to baseline (4942.17 pg/mL), then decreased to 4629.31 pg/mL at one month. The difference between day 1 and month 1 was statistically significant (P = 0.036). No significant changes were observed in IL-12p70 (P = 0.346), IL-6 (P = 0.069), TNF-α (P = 0.157), or IFN-γ (P = 0.058) during the follow-up period (**Table 4**). Subgroup analyses revealed no statistically significant changes in inflammatory cytokines in either the PHPT or SHPT groups across the follow-up period (**Supplementary Table 5**, **6**).

**Table 4:** Changes in inflammatory cytokines before and after ablation.

Time Point	IL-12p70 (pg/mL)	IL-6 (pg/mL)	TNF-α (pg/mL)	IFN-γ (pg/mL)	CRP (pg/mL)
Pre-ablation	2.46 (0.91, 7.26)	3.21 (3.21, 5.42)	33.88 (13.4, 81.71)	0.15 (0.15, 0.32)	4942.17 (3369.64, 11750.55)
Day 1 Post-ablation	0.91 (0.91, 2.37)	3.21 (3.21, 3.21)	56.52 (13.4, 86.02)	0.15 (0.15, 0.15)	5201.34 (4290.57, 12499.18)
Month 1 Post-ablation	0.91 (0.91, 3.85)	3.21 (3.21, 3.21)	13.40 (13.40, 67.99)	0.15 (0.15, 0.15)	4629.31 (3360.48, 5392.32)
P(overall)	0.346	0.069	0.157	0.058	0.029*
P1†	—	—	—	—	0.171
P2‡	—	—	—	—	0.036*
P3§	—	—	—	—	0.136

†P1: Pre-ablation vs. Day 1 Post-ablation, ‡P2: Day 1 Post-ablation vs. Month 1 Post-ablation, §P3: Pre-ablation vs. Month 1 Post-ablation. *P < 0.05 is considered statistically significant.

IL-12p70, interleukin-12p70; IL-6, interleukin-6; TNF-α, tumor necrosis factor-α; IFN-γ, interferon-γ; CRP, C-reactive protein. Biomarker levels in SHPT patients may be influenced by renal function.

### Complications

Two patients (10.5%) developed transient hoarseness after MWA. One patient recovered spontaneously within 1 month, and the other achieved full vocal recovery at 4 months. No cases of severe postoperative hypocalcemia were observed in this cohort.

## Discussion

This preliminary study analyzes the early systemic effects of ultrasound-guided MWA in patients with HPT, focusing on endocrine, skeletal, and inflammatory pathways. Serum iPTH and calcium declined markedly by day 1 and remained within normal range at one month. Bone formation markers showed a sequential rise, with PINP increasing early and BALP following thereafter, while total ALP decreased. FGF-23 levels dropped significantly, and the inflammatory profile remained stable, with only a transient CRP elevation resolving by one month. The procedure demonstrated good short-term safety, with no severe hypocalcemia and only two cases of transient hoarseness. These findings suggest that MWA not only provides rapid biochemical correction but may also promote early skeletal remodeling and modest inflammatory relief without triggering sustained immune activation.

The endocrine response was characterized by a rapid reduction in serum iPTH and calcium by day 1, with iPTH partially rebounding at one month while remaining normal. This pattern reflects effective ablation of hyperfunctioning HPT lesion and subsequent stabilization of residual hormonal activity. Despite the short follow-up period, this pattern closely parallels the early biochemical normalization observed after parathyroidectomy, supporting the potential of MWA to achieve comparable endocrine control in appropriately selected patients (14, 23–25). Subgroup analyses showed a more consistent hormonal response in PHPT, with SHPT patients exhibiting similar trends but without statistical significance—likely due to small sample size and underlying disease complexity.

Skeletal biomarkers followed a biologically coherent matrix-to-mineralization sequence. PINP—recognized by the IOF/IFCC and major clinical guidelines as the reference marker for bone formation—showed a significant increase by one month, reflecting enhanced type I collagen synthesis and activation of osteoblastic activity (26, 27). BALP, though not significantly elevated overall, rose markedly from day 1 to month 1, indicating progression into early mineralization, consistent with the known delay between matrix production and mineral deposition. In contrast, total ALP declined from baseline despite upward trends in bone-specific markers; given its hepatic contribution, total ALP may underestimate skeletal formation and should be interpreted cautiously (28). Bone resorption markers, CTX-I, TRACP-5b and PYD showed no significant changes but trended downward, suggesting a mild early shift toward anabolic balance as PTH levels normalize. This remodeling re-equilibration mirrors findings in post-parathyroidectomy cohorts, where turnover normalizes before measurable gains in bone mineral density occur (29–32). Similar trends were also observed in MWA-treated PHPT populations, with studies reporting significant declines in bone turnover markers and increases in lumbar spine and femoral neck bone mineral density at 12–24 months after ablation, reinforcing the skeletal benefit of early PTH correction through MWA (33, 34). Subgroup analysis showed more pronounced skeletal changes in PHPT patients, with significant PINP elevation and FGF-23 reduction. In SHPT patients, although changes were not statistically significant, trends in PINP, BALP, and FGF-23 were directionally consistent, suggesting biological responsiveness that may have been underestimated due to limited sample size. For SHPT patients with impaired renal function, observed changes in bone turnover and inflammatory markers may reflect both the effects of MWA and underlying renal impairment. Due to the limited number of SHPT patients, stratified statistical analyses by renal function were not performed, and these findings should be interpreted with caution. This highlights the need for larger SHPT cohorts to clarify post-ablation skeletal dynamics.

At one month post-ablation, a notable reduction in FGF-23 was observed, which was similar to the decreasing after surgery (35, 36). As a hormone primarily secreted by osteocytes and osteoblasts, FGF-23 is regulated by iPTH and calcium homeostasis. Its decline indicates effective suppression of PTH-driven skeletal signaling and possible downregulation of high-turnover bone remodeling. Furthermore, elevated FGF-23 has been independently associated with left ventricular hypertrophy and cardiovascular mortality, especially in CKD populations (37). Therefore, the observed reduction may not only reflect endocrine improvement but also represent a mechanistic bridge toward cardiovascular protection—a hypothesis warranting future prospective validation.

Inflammatory activity remained largely stable following MWA. While no significant changes were observed in IL-12p70, IL-6, TNF-α, or IFN-γ levels, CRP showed a statistical decline by one month (P = 0.036), indicating a reduction in systemic inflammatory burden. The absence of cytokine shifts may reflect the chronic, low-grade nature of HPT-associated inflammation, where subtle immune dysregulation is less readily captured by individual cytokine assays. In addition, the short follow-up window, modest sample size, and potential peri-procedural confounders—such as transient inflammatory response to ablation—may further limit cytokine responsiveness. As a hepatic acute-phase protein with high sensitivity and temporal resolution, CRP is better suited to detecting short-term changes of inflammatory tone. Notably, the decline in CRP paralleled the sequential rise in bone formation markers (PINP and BALP), suggesting a possible link between endocrine correction, skeletal remodeling, and immune milieu improvement. This trajectory resembles post-parathyroidectomy patterns, where normalization of PTH is associated with reduced systemic inflammation, including improvements in neutrophil-to-lymphocyte and platelet-to-lymphocyte ratios (38–41). Consistent with our findings, Ren et al. also reported significantly lower postoperative CRP levels in SHPT patients undergoing radiofrequency ablation compared with parathyroidectomy (P = 0.02), highlighting that minimally invasive ablation may attenuate the acute-phase response relative to surgical resection (42). No significant inflammatory changes were observed in either subgroup.These findings support the potential anti-inflammatory benefit of effective HPT correction via MWA, although longer-term data are needed.

MWA demonstrated a favorable safety profile, with only two cases of transient hoarseness and no severe hypocalcemia. This likely reflects standardized protocols and effective hydrodissection technique. The consistent improvement across endocrine, skeletal, and inflammatory markers highlights MWA as a promising minimally invasive option. However, this study has a few limitations. First, the sample size was small, which limits the statistical power and generalizability of the findings. Second, the follow-up period was limited to one month, preventing evaluation of the long-term durability of skeletal and inflammatory improvements. Third, biomarker trajectories were not stratified by disease subtype (PHPT vs SHPT), potentially obscuring differential responses. SHPT patients’ biomarker changes may be influenced by renal function, and no THPT patients were included, limiting generalizability to these subgroups. In addition, this pilot focused on short-term biochemical and immunometabolic responses rather than symptom–biomarker coupling, and standardized patient-reported outcomes were not prespecified. To determine whether the early post-MWA biological responses translate into sustained improvements in bone mineral density, symptom relief, and cardiovascular risk reduction, larger, multicenter prospective studies with extended follow-up and surgical comparators that include standardized patient-reported outcomes are warranted.

## Conclusion

MWA is a safe and effective option for patients with HPT, offering rapid biochemical correction and early changes of skeletal and inflammatory pathways. These results provide preliminary evidence of early systemic biomarker responses beyond local lesion control and highlight the need for longer-term studies to confirm durability and clinical impact.

## Data Availability

The raw data supporting the conclusions of this article will be made available by the authors, without undue reservation.
