# Supplementary material for: Early endocrine, bone, and inflammatory responses to microwave ablation for hyperparathyroidism: preliminary study
Source: Front Endocrinol (Lausanne). 2026 Jul 7;17:1763008. doi: 10.3389/fendo.2026.1763008 (PMC13385488; doi:10.3389/fendo.2026.1763008)
Supplement: Supplementary file 1 [file Table1.doc]

**Supplementary Table 1.** Changes in Parathyroid-Related Laboratory Parameters Before and After Ablation in PHPT patients

| Time Point | iPTH  (pg/mL) | Calcium  (mmol/L) | Phosphorus  (mmol/L) | ALP  (U/L) |
| --- | --- | --- | --- | --- |
| Pre-ablation | 146.7 (111.3, 197.5) | 2.83±0.17 | 0.87 (0.82, 0.94) | 99 (63-111) |
| Day 1 Post-ablation | 18.5 (5.5, 46.6) | 2.46±0.19 | 1.04 (0.86, 1.14) | 102 (65-104) |
| Month 1 Post-ablation | 80.1 (47.4, 95.3) | 2.57±0.43 | 1.15 (1.01, 1.23) | 81 (64-105) |
| P(overall) | <0.001* | 0.001* | 0.010* | 0.411 |
| P1† | 0.001* | 0.001* | 0.177 | — |
| P2‡ | 0.002* | 0.593 | 0.103 | — |
| P3§ | 0.001* | 0.030* | 0.016* | — |

†P1: Pre-ablation vs. Day 1 Post-ablation, ‡P2: Day 1 Post-ablation vs. Month 1 Post-ablation, §P3: Pre-ablation vs. Month 1 Post-ablation. *P < 0.05 is considered statistically significant.

Note: ALP=alkaline phosphatase; iPTH=intact parathyroid hormone.

## Supplementary Table 2. Changes in Parathyroid-Related Laboratory Parameters Before and After Ablation in SHPT patients

| Time Point | iPTH  (pg/mL) | Calcium  (mmol/L) | Phosphorus  (mmol/L) | ALP  (U/L) |
| --- | --- | --- | --- | --- |
| Pre-ablation | 665.1 (379.7, 1429.1) | 2.61±0.15 | 1.66 (1.16, 2.15) | 142 (82-222) |
| Day 1 Post-ablation | 104.6 (39.5, 608.2) | 2.21±0.24 | 1.66 (1.25, 2.03) | 111 (67-186) |
| Month 1 Post-ablation | 123.0 (76,2, 250.0) | 2.54±0.18 | 1.34 (1.08, 2.03) | 82 (69-183) |
| P(overall) | 0.074 | 0.015* | 0.819 | 0.022* |
| P1† | — | 0.043* | — | 0.043* |
| P2‡ | — | 0.043* | — | 0.686 |
| P3§ | — | 0.500 | — | 0.043* |

†P1: Pre-ablation vs. Day 1 Post-ablation, ‡P2: Day 1 Post-ablation vs. Month 1 Post-ablation, §P3: Pre-ablation vs. Month 1 Post-ablation. *P < 0.05 is considered statistically significant.

Note: ALP=alkaline phosphatase; iPTH=intact parathyroid hormone.

**Supplementary Table 3.** Changes in Bone Metabolism Markers Before and After Ablation in PHPT patients

| Time Point | PINP  (pg/mL) | BALP  (ng/mL) | PYD  (ng/mL) | CTX-I  (ng/mL) | TRACP-5b  (mIU/mL) | OPG  (pg/mL) | OT-BGP  (pg/mL) | FGF-23  (pg/mL) |
| --- | --- | --- | --- | --- | --- | --- | --- | --- |
| Pre-ablation | 11715.81±16526.01 | 0.68  (0.37, 2.25) | 5.48  (1.50,36.08) | 6.74 ± 4.00 | 3.44 ± 2.33 | 67.88  (11.35, 120.50) | 5499.16  (2151.61-14603.60) | 0.09  (0.01, 7.06) |
| Day 1  Post-ablation | 10389.13±10433.24 | 0.49  (0.37, 0.95) | 1.50  (1.50, 11.25) | 5.50 ± 3.73 | 3.24 ± 2.56 | 29.16  (8.97, 104.65) | 3675.72  (2563.14-20431.48) | 0.01  (0.01, 2.84) |
| Month 1  Post-ablation | 22262.53±7833.60 | 0.84  (0.65, 10.67) | 1.50  (1.50, 5.58) | 5.71 ± 4.65 | 2.55 ± 2.45 | 41.35  (8.97, 114.21) | 4744.00  (552.07-10189.70) | 0.01  (0.01, 0.01) |
| P(overall) | <0.001* | 0.052 | 0.113 | 0.538 | 0.368 | 0.079 | 0.017* | 0.009* |
| P1† | 0.213 | — | — | — | — | — | 0.771 | 0.374 |
| P2‡ | 0.002* | — | — | — | — | — | 0.014* | 0.043* |
| P3§ | 0.013* | — | — | — | — | — | 0.217 | 0.012* |

## †P1: Pre-ablation vs. Day 1 Post-ablation, ‡P2: Day 1 Post-ablation vs. Month 1 Post-ablation, §P3: Pre-ablation vs. Month 1 Post-ablation. *P < 0.05 is considered statistically significant

## Note: PYD=pyridinoline; BALP=bone-specific alkaline phosphatase; PINP=procollagen type I N-terminal propeptide; CTX-I=C-terminal telopeptide of type I collagen; TRACP-5b=tartrate-resistant acid phosphatase 5b; OPG=osteoprotegerin; OT-BGP=osteocalcin; and FGF-23=fibroblast growth factor-23.

## Supplementary Table 4. Changes in Bone Metabolism Markers Before and After Ablation in SHPT patients

| Time Point | PINP  (pg/mL) | BALP  (ng/mL) | PYD  (ng/mL) | CTX-I  (ng/mL) | TRACP-5b  (mIU/mL) | OPG  (pg/mL) | OT-BGP  (pg/mL) | FGF-23  (pg/mL) |
| --- | --- | --- | --- | --- | --- | --- | --- | --- |
| Pre-ablation | 1725.40±10594.72 | 2.51  (0.60, 6.43) | 13.38  (6.24, 29.62) | 11.24 ± 6.00 | 6.39 ± 1.84 | 64.39  (27.29, 267.89) | 35299.83  (5483.33-45447.46) | 0.09  (0.01, 5.45) |
| Day 1  Post-ablation | 18490.41±12199.07 | 2.43  (0.89, 7.16) | 12.04  (1.50, 24.55) | 12.56 ± 6.39 | 5.21 ± 1.10 | 2524.43  (82.77, 429.40) | 32422.49  (2171.44-44976.21) | 2.32  (0.01, 8.88) |
| Month 1  Post-ablation | 23394.04±3735.74 | 1.65  (0.83, 10.62) | 1.50  (1.50, 42.61) | 10.69 ± 5.39 | 5.00 ± 3.98 | 39.86  (14.67, 453.06) | 33728.93  (10492.14-45874.37) | 0.01  (0.01, 1.79) |
| P(overall) | 0.143 | 0.949 | 0.420 | 0.247 | 0.819 | 0.549 | 0.819 | 0.717 |
| P1† | — | — | — | — | — | — | — | — |
| P2‡ | — | — | — | — | — | — | — | — |
| P3§ | — | — | — | — | — | — | — | — |

## †P1: Pre-ablation vs. Day 1 Post-ablation, ‡P2: Day 1 Post-ablation vs. Month 1 Post-ablation, §P3: Pre-ablation vs. Month 1 Post-ablation. *P < 0.05 is considered statistically significant

## Note: PYD=pyridinoline; BALP=bone-specific alkaline phosphatase; PINP=procollagen type I N-terminal propeptide; CTX-I=C-terminal telopeptide of type I collagen; TRACP-5b=tartrate-resistant acid phosphatase 5b; OPG=osteoprotegerin; OT-BGP=osteocalcin; and FGF-23=fibroblast growth factor-23.

**Supplementary Table 5.** Changes in Inflammatory Cytokines Before and After Ablation in PHPT patients

| Time Point | IL-12p70  (pg/mL) | IL-6  (pg/mL) | TNF-α  (pg/mL) | IFN-γ  (pg/mL) | CRP  (pg/mL) |
| --- | --- | --- | --- | --- | --- |
| Pre-ablation | 2.19  (0.91, 10.64) | 3.21  (3.21, 3.76) | 15.27  (13.4, 81.13) | 0.15  (0.15, 0.42) | 4797.14  (3308.6, 11959.64) |
| Day 1 Post-ablation | 0.91  (0.91, 8.13) | 3.21  (3.21, 3.21) | 56.65  (13.4, 87.08) | 0.15  (0.15, 0.15) | 5325.33  (4163.86, 12850.81) |
| Month 1 Post-ablation | 0.91  (0.91, 3.97) | 3.21  (3.21, 3.21) | 13.4  (13.4, 39.49) | 0.15  (0.15, 0.15) | 4422.55  (3314.09, 5431.64) |
| P(overall) | 0.558 | 0.061 | 0.110 | 0.068 | 0.109 |
| P1† | — | — | — | — | — |
| P2‡ | — | — | — | — | — |
| P3§ | — | — | — | — | — |

†P1: Pre-ablation vs. Day 1 Post-ablation, ‡P2: Day 1 Post-ablation vs. Month 1 Post-ablation, §P3: Pre-ablation vs. Month 1 Post-ablation. *P < 0.05 is considered statistically significant.

Note: IL-12p70=interleukin-12p70; IL-6=interleukin-6; TNF-α=tumor necrosis factor-α; IFN-γ=interferon-γ; CRP=C-reactive protein.

**Supplementary Table 6.** Changes in Inflammatory Cytokines Before and After Ablation in SHPT patients

| Time Point | IL-12p70  (pg/mL) | IL-6  (pg/mL) | TNF-α  (pg/mL) | IFN-γ  (pg/mL) | CRP  (pg/mL) |
| --- | --- | --- | --- | --- | --- |
| Pre-ablation | 2.46  (0.91, 3.3) | 3.21  (3.21, 23.23) | 63.31  (23.64, 91.73) | 0.15  (0.15, 0.75) | 4942.17  (3028.89, 15342.35) |
| Day 1 Post-ablation | 0.91  (0.91, 1.86) | 3.21  (3.21, 20.99) | 56.05  (23.02, 78.68) | 0.15  (0.15, 0.90) | 5201.34  (4413.77, 5563.22) |
| Month 1 Post-ablation | 0.91  (0.91, 5.58) | 3.21  (3.21, 23.21) | 68.78  (13.4, 89.2) | 0.15  (0.15, 0.15) | 4764.13  (3505.54, 5245.99) |
| P(overall) | 0.584 | 0.607 | 0.779 | 0.368 | 0.247 |
| P1† | — | — | — | — | — |
| P2‡ | — | — | — | — | — |
| P3§ | — | — | — | — | — |

†P1: Pre-ablation vs. Day 1 Post-ablation, ‡P2: Day 1 Post-ablation vs. Month 1 Post-ablation, §P3: Pre-ablation vs. Month 1 Post-ablation. *P < 0.05 is considered statistically significant.

Note: IL-12p70=interleukin-12p70; IL-6=interleukin-6; TNF-α=tumor necrosis factor-α; IFN-γ=interferon-γ; CRP=C-reactive protein.
